# Injuries in Recreational Footballers and the Severe Consequences of Player-to-Player Contact: A Prospective Observational Study

**DOI:** 10.7759/cureus.21758

**Published:** 2022-01-31

**Authors:** Davut Tekyol, Gürkan Akman, Sinem Doğruyol, İlker Akbaş

**Affiliations:** 1 Emergency Medicine, University of Health Sciences, Haydarpasa Numune Training and Research Hospital, Istanbul, TUR; 2 Emergency Medicine, Kartal Dr Lutfi Kirdar City Hospital, Istanbul, TUR; 3 Emergency Medicine, Sutcu Imam University, Medical Faculty, Kahramanmaras, TUR

**Keywords:** blunt thoracic trauma, craniofacial trauma, sports injury, player-to-player contact, football

## Abstract

Introduction

Football is the most popular sport in the world with its wide audience and important economic effects. This game is mainly played by professional football players, it is also an activity that is frequently played by non-professionals. Although members of the public mostly engage in this sport as a hobby and to develop a healthy lifestyle, vital injuries, such as head and thorax trauma can also occur during these recreational activities. In this study, our aim was to identify these severe traumas, investigate their causes and mechanisms, and make suggestions to reduce trauma among recreational football players.

Methods

This prospectively designed study included players aged over 14 years who presented to the emergency department with an injury incurred during a recreational football match activity. The demographic characteristics of the patients, warm-up status, match conditions, field conditions, and injury mechanisms, as well as post-injury outcomes, were recorded and analyzed with appropriate statistical methods.

Results

There were 167 patients included in the study and 140 of these patients were eligible for the study. We identified 45 cases with poor outcomes such as pneumocephalus, rib fractures, pneumothorax, Achilles tendon rupture, and bone fractures. (32.1%). Factors that could have an effect on poor outcomes were determined as a pre-match warm-up, pre-match sleep duration, and suitability of equipment. Player-to-player contact was determined as a mechanism of head and thorax trauma.

Conclusions

It may be beneficial to inform recreational football players about how player-to-player contact can cause head and thoracic trauma and establish a database of sports injuries in hospitals.

## Introduction

Football is a team game and the most popular sport in the world with its wide audience and important economic impact [1]. This popularity has carried football far beyond being a sport played only by professional athletes. In many countries, people watch football matches on television or in stadiums as fans and play football for recreation. In Turkey, places, where recreational football (RF) is most commonly played, are traditionally called astroturf pitches or artificial turf fields. People play on these fields specifically designed for RF. Hundreds of thousands of people play football on approximately 10,000 fields across Turkey [2]. Although there are many studies on professional football players in the literature, the number of publications examining non-professional matches such as RF is less. Considering that such matches are held in many countries, they actually constitute a large part of all football matches.

It is known that playing football has high cardiovascular, metabolic and psychiatric positive effects [3]. However, even if it is a sports activity people engage in for the purpose of enjoyment and supporting a healthy lifestyle, the incidence of injury in football is higher than in other sports [4]. Football requires power, speed, and endurance [5]. RF players are people who continue their professional lives on other days of the week and do not have the opportunity to train continuously. In addition, in terms of preparation for the match, both their condition level and technical knowledge are lower compared to professional football players [6]. This further increases the risk of football-related injuries among RF players. These injuries range from minor soft tissue trauma to life-threatening trauma. The inability of players to continue their daily lives results in the loss of labor and time, which can lead to disabilities [7].

Due to their complex nature, examining and understanding injuries is the most important part of the dynamic process for injury prevention interventions [8]. The management of football player injuries according to standard trauma algorithms makes it difficult to create a database on this subject. In this study, our aim was to investigate injuries in RF matches and determine the player- and environment-related factors as mechanisms of serious injuries.

## Materials and methods

Study design

This study was designed prospectively and conducted in a tertiary hospital from July 1, 2021, to December 31, 2021. All the patients included in the study were informed about the study and its procedures in advance. Written informed consent was obtained from the patients, who were found eligible for the study, at the time of admission to the emergency department. The study was approved by the Clinical Research Ethics Committee of Istanbul Haydarpaşa Numune Training and Research Hospital with the file number HNEAH-KAEK 2021/KK/60, dated March 1, 2021.

Patient selection

Patients aged 14 years and older with RF-related injuries who presented to the emergency department were evaluated for their eligibility for the study. Cases that were passed 24 hours after the trauma and those who did not proper the study criteria were excluded from the study.

Outcomes

The patients' demographic data, height/weight information, patient characteristics before and during the match, field conditions, trauma mechanism and localization, trauma-related injuries and their effect on the match, post-injury treatment, and disability data were recorded. Body mass index (BMI) was calculated. 

Mechanisms of trauma were classified as contact (player-to-player or player-to-object) and non-contact (falling, twisting/turning of an extremity, and stretching). According to the findings obtained in the examination after the injury, types of injury were collected in six groups namely, contusion/abrasion, sprain/strain, laceration, swelling/hematoma, fracture, and dislocation. While identifying the injured body regions, the areas of primary trauma were noted.

Poor outcomes due to injury accepted as the need for referral to the hospital by ambulance, presence of large superficial incisions and tissue loss, need for a splint/elastic bandage, need for follow-up in the emergency department, need for hospitalization and surgical intervention, and injury resulting workday/schoolday loss for more than one week. The occurrence of at least one of these conditions in a patient was defined as a poor outcome.

Statistical analysis

All statistical analyses were performed using Statistical Package for the Social Sciences (SPSS) (IBM Corp. Released 2011. IBM SPSS Statistics for Windows, Version 20.0. Armonk, NY: IBM Corp). The conformity of the data to the normal distribution was examined with the Kolmogorov-Smirnov test. Percentage frequency was used while expressing categorical data, while mean (± standard deviation) and median (Inter quantile range-IQR) values ​​were used to describe continuous data. The Pearson's χ2-test was used for calculating the differences between categorical variables of two patient groups. The Student’s t-test was conducted for the comparison of continuous data between two groups. The multivariate logistic regression analysis was performed to examine factors that might have an effect on the incidence of injuries with poor outcomes. When explaining the results of multivariate logistic regression analysis odds ratio (OR) and 95% confidence level (CI) was used. The results were expressed at the 95% CI and p<0.05 significance level.

## Results

During the study, 167 patients admitted to the emergency department due to RF-related injury were evaluated in terms of eligibility for the study, and 140 were included in the sample. All the patients were male, and their mean age was 24.6±8.6 (range:14-55) years. The height, weight, and BMI values ​​of the patients are shown in Table 1.

**Table 1:** Baseline and before-match patient characteristics Values expressed as numbers with percentages or mean ± standard deviation BMI: Body Mass Index

	Patients (n=140)
Age groups	
14-25 years	83 (59.3%)
25-35 years	39 (27.9%)
35-45 years	15 (10.7%)
45-55 years	3 (2.1%)
Height (cm)	174.0±6.9
Weight (kg)	73.7±11.7
BMI (kg/m^2^)	24.6±3.1
Dominant foot	
Right	67 (47.9%)
Left	73 (52.1%)
History of disability/injury	
None	92 (65.7%)
0-12 months	38 (27.1%)
>12 months	10 (7.1%)
History of regular exercise	
Absent	93 (66.4%)
Present	47 (33.6%)
Sleep duration (within the last 24 hours)	
0-4 hours	22 (15.7%)
4-8 hours	81 (57.9%)
8-12 hours	37 (26.4%)
Last meal	
0-2 hours	38 (27.1%)
2-4 hours	66 (47.1%)
>4 hours	36 (25.7%)

**Figure 1:**
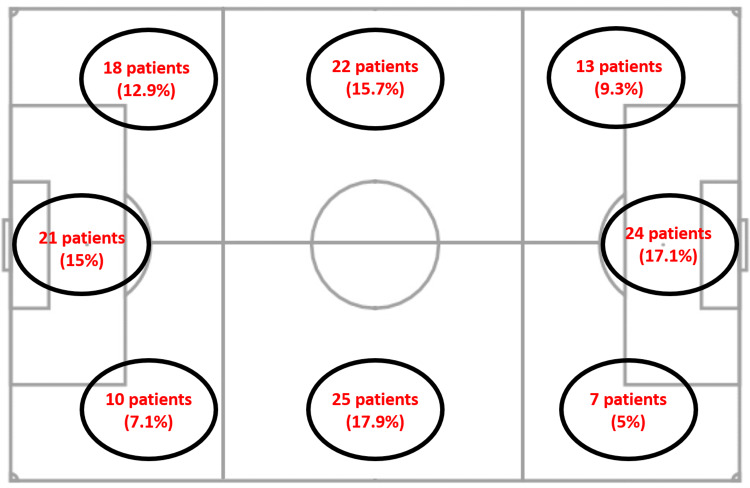
Figure showing the distribution of the patients presenting with injuries according to their positions on artificial turf field

The sports-related characteristics of the patients and their before-match habits are given in Table 1. When the organization hours of the matches on football fields were examined, it was determined that 90 (64.3%) matches were played at or after 6 p.m. and 50 (35.7%) matches were played before 6 p.m. The air temperature data at the time the matches were played were also examined, and it was determined that only one match was held at sub-zero temperature while 17.9% of the matches were held at 0 to 10 °C, 67.1% at 10 to 20 °C, and 14.3% at 20°C and above. Concerning the properties of the football fields, 89 (63.6%) of the patients stated that they were injured on dry ground, 51 (36.4%) on wet ground, and 13 (9.3%) noted that the ground was bad. While 88 (62.9%) of the patients stated that they wore shoes without spikes during the match, 97 (69.3%) stated that they wore specific equipment for the football match. The distribution of the patients according to their positions during the match is shown in Figure 1.

Table 2 presents the distribution of RF-related injuries according to their mechanisms. When the injured body regions were examined anatomically, the most frequently injured areas were the ankle (19.3%), knee (14.3%), and foot/toes (11.4%) (Table 3).

**Table 2:** Trauma mechanisms Values expressed as numbers with percentages *The Pearson's χ2-test was used for calculating the differences between the categorical variables of these two patient groups.

	All Patients (n=140)		
	With poor outcome (n=45)	Without poor putcome (n=95)	p values
Warm-up status			
None	24 (53.3%)	27 (28.4%)	p=0.004
0-10 minutes	21 (46.7%)	68 (71.6%)	
Mechanism			
Contact			
Player-to-player	24 (53.3%)	34 (35.8%)	p=0.009
Player-to-object	7 (15.6%)	9 (9.5%)	
Non-contact			
Twisting/turning	3 (6.7%)	31 (32.6%)	
Fall	11 (24.4%)	18 (18.9%)	
Stretching	0 (0.0%)	3 (3.2%)	

**Table 3:** Data on the primary body regions affected by injury Values expressed as numbers with percentages

	Patients (n=140)
Body region	
Ankle	27 (19.3%)
Knee	20 (14.3%)
Foot/toes	16 (11.4%)
Shoulder	15 (10.7%)
Hand/fingers	14 (10.0%)
Head/face	12 (8.6%)
Thorax	8 (5.7%)
Lower leg	6 (4.3%)
Thigh	5 (3.6%)
Forearm	5 (3.6%)
Hip/groin	5 (3.6%)
Lumbar spine	3 (2.1%)
Wrist, elbow	2 (1.4%)
Neck	2 (1.4%)

According to the injury-related findings, 51 patients (36.4%) were injured in the first half of the match and 89 patients (63.6%) in the second half. Sixty-one (43.6%) of the patients continued the match after injury. The distribution of injury types is shown in Table 4. When the post-injury treatment modalities were examined, splints/elastic bandages were applied to 50 patients (35.7%), tissue injuries requiring sutures and dressings were seen in 11 (7.9%), andinjuries requiring surgical treatment were observed in 13 (9.3%).

**Table 4:** Distribution of injury types Values expressed as numbers with percentages

	Patients (n=140)
Injury type	
Swelling/hematoma	53 (37.9%)
Sprain/strain	34 (24.3%)
Fracture	31 (22.1%)
Contusion/abrasion	13 (9.3%)
Laceration	7 (5.0%)
Dislocation	2 (1.4%)

The time taken unable to play sports due to injury was ≥ seven days for 59 (42.1%) of the patients, 8-30 days for 30 (21.4%), and 31 days to three months for 26 (18.6%). The Pearson's χ2-test was used for calculating the differences between the distribution of time taken unable to play sports after injury by age groups. The time taken unable to play sports after injury was significantly higher among the patients aged 35-45 years compared to the other age groups (p=0.038). The time taken off work/school due to injury was one day for 16 (11.4%) of the patients, two to seven days for 32 (22.9%), and eight to 30 days for 19 (13.6%).

A total of eight patients had thorax-related injuries. The relationship between injured body regions and trauma mechanisms was calculated with Pearson's χ2-test , and it was observed that the most common trauma mechanism of patients with thorax injuries was player-to-player contact (PPC) (n=7, 87.5%), which was statistically significant (p=0.001). In the patient group with thorax injuries, the rate of those taking time off work/school for eight to 30 days was statistically significantly higher compared to the other injury localizations (p=0.000). Two of the patients with thorax trauma had rib fractures and pneumothorax, and both underwent tube thoracostomy.

Among our cases, head/face trauma was seen in 12 patients, of whom nine had facial or nasal injury. The results of the Pearson's χ2-test showed that; the mechanism of trauma was mostly PPC for this patient group (n= 10, 83.3%) (p=0.001). One of the patients with head/face trauma had pneumocephalus due to a skull fracture. The duration of disability due to head/face trauma was calculated with Student’s t-test. We did not find a significantly different result compared to the other trauma localizations (p=0.882).

Forty-five (32.1%) patients had poor outcomes. The multivariate logistic regression analysis was conducted to examine independent factors related to the pre-match data and personal data (before and during the match) that could have an effect on poor outcomes. Among these factors, pre-match warm-up status [odds ration (OR): 2.59; 95%, confidence interval (CI):1.11-6.03, p=0.027], pre-match sleep duration (OR :3.46; 95%, CI:1.29-9.24, p=0.013), specific equipment use (OR : 0.42; 95% CI:0.18-0.98, p=0.046) and PPC (OR:2.55; 95% CI: 1.42-5.68, p=0.022), were found to have effects on outcomes. The frequency of poor outcomes was 23.6% in patients who had warmed up for 0-10 minutes before the match, it was 47.1% among patients who did not engage in any warm-up, indicating a statistically significant difference (p=0.004).

## Discussion

The emergency departments are the first places to which individuals injured in RF matches refer. The first intervention and examination processes of these patients are not different from other trauma cases. However, RF is a sport with a high risk of injury. There are many factors that affect the formation of trauma in this sport, and therefore examining the types and characteristics of RF trauma can help prevent future injuries. Although RF matches are held for entertainment purposes, the incidence of serious injuries is at an alarming level. The main purpose of our study was to examine the characteristics of patients presenting to the emergency department with RF-related trauma and establish a regular data recording system. Contrary to expectations, we detected a high probability of encountering not only extremity injuries but also critical thorax and head trauma cases.

In a previous study, Kordi et al. compared the characteristics of injuries in matches played in a dirt field and artificial turf field [9]. The authors reported that the most common injury mechanism was PPC. Similarly, in our study, more than half the patients were injured due to a collision with another player; i.e., PPC.

In our study, the most frequently injured body region was the lower extremity, which was seen in nearly 80% of the patients. The ankle and knee were the most affected areas in this region. This is consistent with the literature, with the ankle and knee being the unchanging localization of football injuries, whether professional or amateur [6,10,11]. In our study, different from the literature, we also detected a high frequency of thorax injuries. In the literature examining football injuries, thorax injuries are generally included in multiple or trunk injuries [12,13]. Padegimas et al. reported that the rate of emergency department presentations was high in the presence of sports-related thorax injuries [14]. In our study, almost all the patients with thorax injuries had fractures of the thoracic osseous structures. Some of these patients required tube thoracostomy and were hospitalized for this procedure.

In the current study, we also examined the relationship between traumatized body regions and the severity of injuries and injury mechanisms and found that PPC was the most common injury mechanism in patients with severe clinical findings, such as pneumothorax and pneumocephalus. In the literature, Levy et al. stated that more than 80% of the concussion injuries reported in football matches were caused by PPC [15]. At first glance, PPC can appear to cause simple injuries compared to other mechanisms resulting in general body trauma. However, in our study, the main mechanism in patients with serious injuries was PPC, which is consistent with the literature. Therefore, RF players should be made aware of the possible serious outcomes of this type of injury.

According to the criteria used in our study, 30% of injuries were evaluated to result in poor outcomes. In a study by Beijsterveldt et al., examining injuries among amateur football players, the authors categorized injury severity according to recovery time and found that the duration of injury was 8-28 days for their moderate trauma category and >28 days for the severe trauma category, with the mean frequency of injury being around 30-45% [16]. Concerning the similar injury frequency and severity we observed, we can state that although the players in our study did not have any amateur or professional sports background, the severity of trauma to which they were exposed was very high considering that this activity was undertaken for recreational purposes.

In this study, we also examined independent risk factors that could have an effect on poor outcomes. We determined that one of these factors was the pre-match warm-up status of the players.. The trauma clinic was more severe in patients who had not engaged in any warm-up exercise before the match. There are many studies in the literature investigating the effectiveness of warm-up exercises in reducing injuries [17]. Soligard et al., evaluating female football players, stated that a 15-minute warm-up exercise could reduce all injury rates, including serious injuries [18]. Consistent with the literature, in the current study, we observed that even a 10-minute pre-match warm-up exercise was effective in decreasing the frequency of injury.

Limitations

The primary limitation of our study is the small number of patients, which was mainly due to artificial turf fields being closed during the pandemic, resulting in the suspension of RF events. In addition, since there is no database created for recording injuries that occur in these football fields in Turkey, the data we presented only represent the results of a single center. However, even in the presence of small sample size, the number of patients with serious trauma was at a level that cannot be underestimated.

## Conclusions

Although RF is regarded as a hobby, it is also an activity with a high probability of causing severe injuries. The probability of injury among RF players is similar to professional football players. It may be beneficial to warm up before the match, to be more careful in player-to-player contacts, and to use protective equipment for ankle, knee, and foot/toes, which account for almost half of the injuries. In addition, a common diagnostic system in all hospitals can provide clinicians with more useful data. In this way, RF played for pleasure and health purposes can become more enjoyable and safer.
